# Indolo[3,2-*b*]indole-based crystalline hole-transporting material for highly efficient perovskite solar cells†
†Electronic supplementary information (ESI) available: Experimental details and additional supplementary figures. CCDC 1453030. For ESI and crystallographic data in CIF or other electronic format see DOI: 10.1039/c6sc02832b


**DOI:** 10.1039/c6sc02832b

**Published:** 2016-09-05

**Authors:** Illhun Cho, Nam Joong Jeon, Oh Kyu Kwon, Dong Won Kim, Eui Hyuk Jung, Jun Hong Noh, Jangwon Seo, Sang Il Seok, Soo Young Park

**Affiliations:** a Center for Supramolecular Optoelectronic Materials , Department of Materials Science and Engineering , Seoul National University , 1 Gwanak-ro, Gwanak-gu , Seoul , 151-744 , Republic of Korea . Email: parksy@snu.ac.kr; b Division of Advanced Materials , Korea Research Institute of Chemical Technology , 141 Gajeong-Ro, Yuseong-Gu , Daejeon 305-600 , Republic of Korea . Email: jwseo@krict.re.kr; c School of Energy and Chemical Engineering , Ulsan National Institute of Science and Technology (UNIST) , 50 UNIST-gil, Eonyang-eup , Ulsan 689-798 , Republic of Korea . Email: seoksi@unist.ac.kr ; Email: seoksi@krict.re.kr

## Abstract

We have designed and synthesized fluorinated indolo[3,2-*b*]indole (IDID) derivatives as crystalline hole-transporting materials (HTM) for perovskite solar cells. The fluorinated IDID backbone enables a tight molecular arrangement stacked by strong π–π interactions, leading to a higher hole mobility than that of the current HTM standard, *p*,*p*-spiro-OMeTAD, with a spherical shape and amorphous morphology. Moreover, the photoluminescence quenching in a perovskite/HTM film is more effective at the interface of the perovskite with **IDIDF** as compared to that of *p*,*p*-spiro-OMeTAD. As a consequence, the device fabricated using **IDIDF** shows superior photovoltaic properties compared to that using *p*,*p*-spiro-OMeTAD, exhibiting an optimal performance of 19%. Thus, this remarkable result demonstrates IDID core-based materials as a new class of HTMs for highly efficient perovskite solar cells.

## Abstract 2

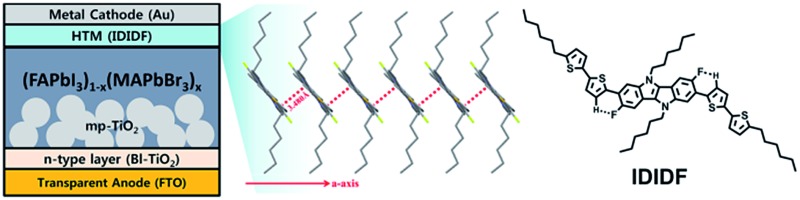
A fluorinated indolo[3,2-*b*]indole (IDID) derivative is prepared as a crystalline hole transporting material for perovskite solar cells. A fluorinated IDID backbone enables a tight molecular stacking by π–π interaction. The device fabricated using IDIDF exhibits a PCE of 19%.

## Introduction

Inorganic/organic lead halide perovskite solar cells (PSCs) have attracted significant attention due to advantages such as low-cost fabrication, low weight, flexibility, and high performance, already surpassing a power conversion efficiency (PCE) of 22%.^1^ Among various device architectures, high efficiencies have been achieved in an n–i–p-type “bilayered” structure, employing a perovskite layer as a light absorber atop a mesoporous (mp)-TiO_2_ scaffold as a n-type selective contact and hole-transporting material (HTM).^1*d*^

In this device configuration, an ideal HTM in perovskite solar cells requires a highest occupied molecular orbital (HOMO) level that is well-matched with the valence band of the perovskite for hole injection, and a high mobility for hole extraction without recombination.^2^ To date, several attempts have been made to develop a best-performing class of HTMs by incorporating ethylenedioxythiophene, cruciform oligothiophene, fused thiophene, pyrene, quinolizinoacridine, and triptycene as good p-type units into a molecular-core structure.^3^ Despite such efforts, so far, triarylamine-based HTMs, including 2,2′,7,7′-tetrakis(*N*,*N*-di-*p*-methoxyphenylamine)-9,9′-spirobifluorene (*p*,*p*-spiro-OMeTAD) and polytriarylamine (PTAA), have been considered to be the most effective for facilitating hole extraction and preventing electron leakage from the perovskite layer toward the electrode.^2^ Very recently, spiro-OMeTAD derivatives offered a superior performance to that of *p*,*p*-spiro-OMeTAD, as a result of simply modifying a position of the methoxy substituents or introducing a fluorene-dithiophene into a spirobifluorene core.^4^

Recently, as a similar p-type analogue to triarylamine, pyrrole-containing heteroacene HTMs based on carbazole, indolocarbazole, and fused indoles have been extensively investigated because of their interesting features:^5^ the low cost of carbazole and its derivatives, a low redox potential due to strong electron-donating properties, a good chemical-environmental stability due to full aromaticity, and the capacity for molecular structural variation by introduction of alkyl groups or functional groups into the nitrogen atom or the outer benzene, which allows for tuned electronic properties, controllable solubility, and controllable molecular packing. For example, a star-shaped triazatruxene derivative containing three indole units combined by one benzene was utilized as a HTM in perovskite solar cells, exhibiting a remarkable PCE over 18%, which is superior to that obtained with *p*,*p*-spiro-OMeTAD.^5*c*^

**Fig. 1:**
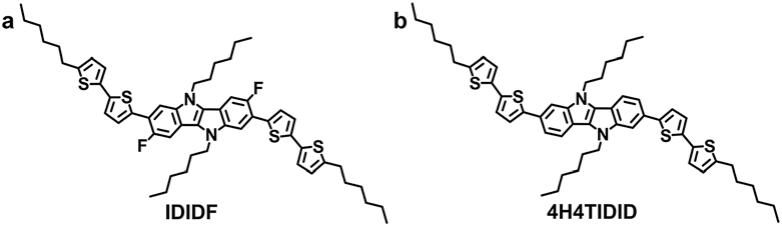
Molecular structure of **IDIDF** (a) and **4H4TIDID** (b).

Among various types of pyrrole-containing heteroacene, attempts have been made to exploit indolo[3,2-*b*]indole (IDID)-based π-conjugated p-type semiconductors in organic light-emitting diodes (OLED), organic field-effect transistors (OFET), and organic photovoltaics (OPV).^6^ Very recently, we demonstrated the high potential of IDID as a p-type backbone unit with its well-designed molecular structure (see Fig. 1b), exhibiting an outstanding field-effect hole mobility of 0.97 cm^2^ V^–1^ s^–1^ in a vacuum-deposited (VD) crystalline film, and versatile processability.^7^ However, the solution-processed spin-coated film showed a somewhat lower field-effect hole mobility (0.18 cm^2^ V^–1^ s^–1^) than that of the VD device, thereby requiring more optimization with respect to the molecular structure for better molecular packing upon aggregation from the concentrated solution. In fact, this is crucial for its use as a high-performance HTM in PSCs.

In this regard, we have introduced a fluorine substituent into the IDID core (at the 3- and 8-positions) to enable an intramolecular interaction with the neighbouring thiophene through C–F···H, generating an extended planar π-conjugated backbone suitable for a p-type HTM (**IDIDF** in Fig. 1a) for PSCs. Its optoelectronic and electrochemical properties were characterized, together with single-crystal analysis. With respect to the hole mobility, the HOMO energy level, and the hole collection capability at the perovskite/HTM interface, **IDIDF** showed a better performance when compared with *p*,*p*-spiro-OMeTAD. As a result, we could demonstrate this new **IDIDF** compound as a best-performing class of HTMs for PSCs by achieving a high efficiency of 19% in this work.

## Results and discussion

**Scheme 1:**
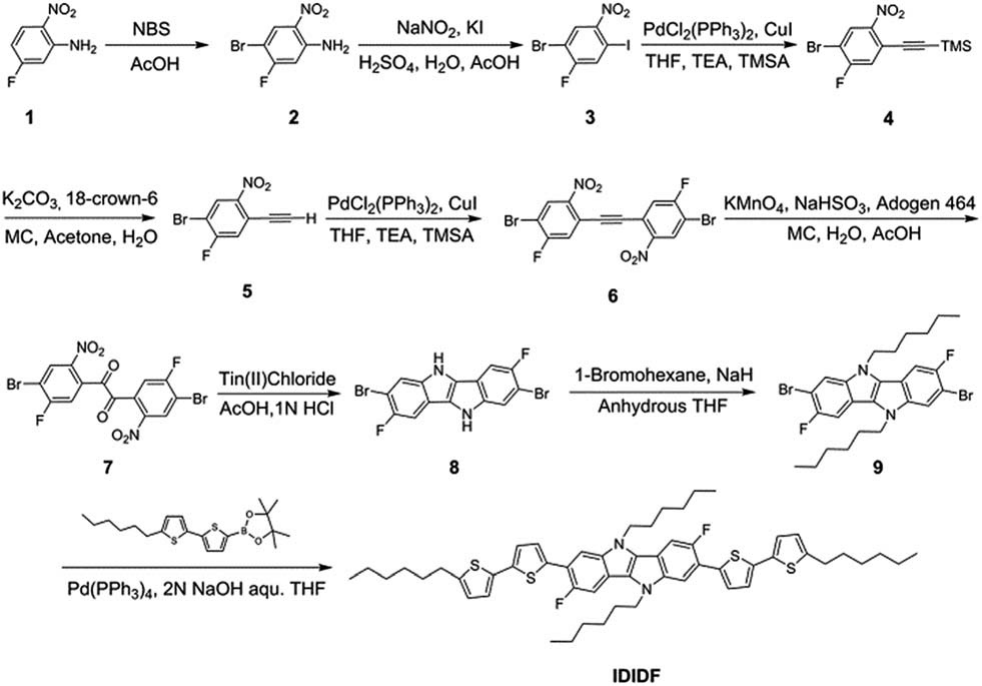
Synthetic route for **IDIDF**.

Fig. 1a depicts the molecular structure of **IDIDF**. As shown in Scheme 1, the synthetic procedure for the fluorinated IDID core, 2,7-dibromo-3,8-difluoro-5,10-dihydroindolo[3,2-*b*]indole (**8**), is essentially the same as that for 2,7-dibromo-5,10-dihydroindolo[3,2-*b*]indole, which was described in our previous report,^7^ but the additional bromination reaction of 5-fluoro-2-nitroaniline (**1**) is preceded. The obtained fluorinated IDID core was sequentially substituted with *n*-hexyl chains at two *N*-positions and a 5-hexyl-2,2′-bithiophene unit at the 2-, 7-position of the fluorinated IDID core by an S_N_2 reaction and Suzuki–Miyaura coupling reaction, respectively. The newly synthesized molecule, **IDIDF**, exhibited an acicular crystal habit, and is highly soluble in common organic solvents. The molecular structure was carefully characterized by ^1^H NMR, ^13^C NMR, elemental analysis, and mass analysis. The detailed synthetic procedure is described in the Experimental section.

**Fig. 2:**
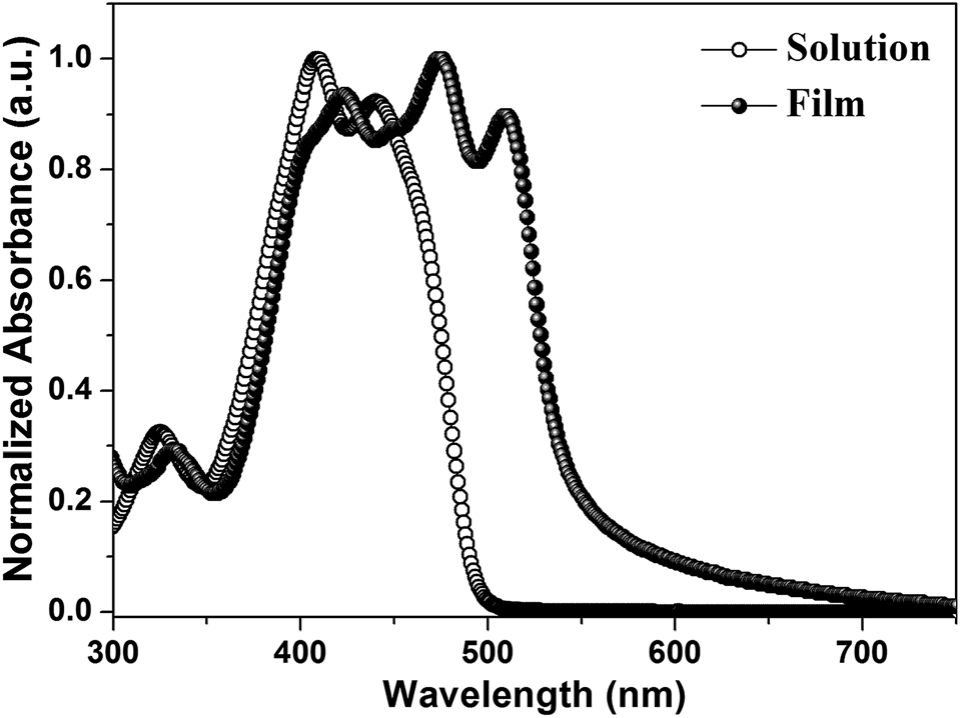
UV-vis absorption spectra of **IDIDF** in a solution (tetrahydrofuran, 1 × 10^–5^ M) and film state.

Fig. 2 displays UV-vis absorption spectra of **IDIDF** in solution and in the film state. In the solution state, the π–π* transition of the absorption band was observed at 440 nm, whereas in the film state, the corresponding π–π* transition was found at 510 nm. Compared to that in solution, a large bathochromic shift of the absorption spectra in the film is clearly observed. In association with this shift, the solution-processed film, with good transparency, exhibited an apparently crystalline nature (see Fig. S1 in the ESI†), which is totally different from many amorphous HTMs, including spiro-OMeTAD for PSCs. As a result, this crystalline feature indicates a highly ordered structure of **IDIDF** molecules, most probably due to strong intermolecular interactions.

**Fig. 3:**
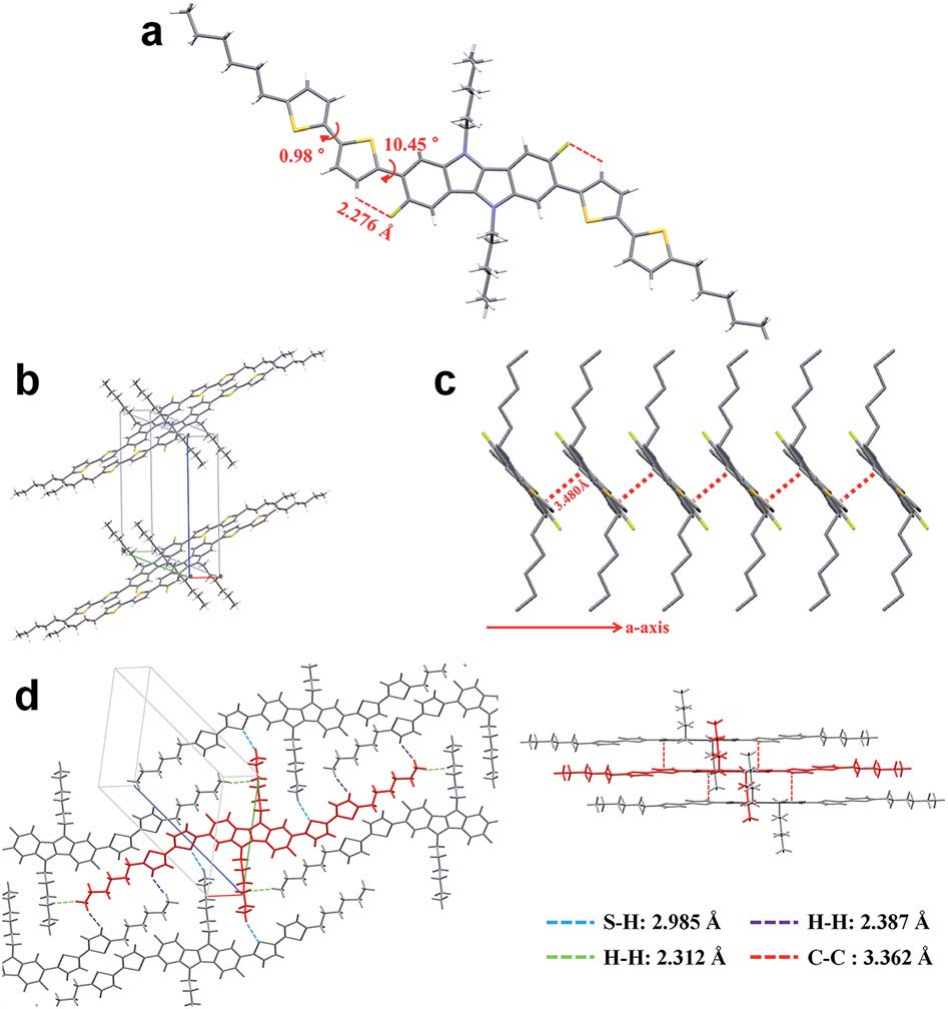
Single-crystal analysis of **IDIDF**.

To fully elucidate the molecular conformation and packing motif of **IDIDF** in the solid state, single-crystal X-ray crystallography was performed (see Fig. 3, S2 and Table S1†). As for the molecular conformation, **IDIDF** exhibited an almost planar conformation with a torsion angle of 10.45° between the fluorinated IDID core and neighbouring thiophene, and a torsion angle of 0.98° between the two thiophenes. More importantly, the F···H distance between the fluorinated IDID core and a neighbouring thiophene was found to be 2.276 Å, which is less than the sum of the van der Waals radii of the fluorine and the hydrogen (=about 2.67 Å).^8^ Thus, the strong intramolecular interaction of C–F···H is one of the key factors reducing the distortion between the IDID core and the neighbouring thiophene, and enhancing the planarity of the elongated molecule. This may enable **IDIDF** to successfully build a single crystal. In contrast, for a molecule that was the same as **IDIDF**, except for F, **4H4TIDID**, we could not obtain a single crystal for identifying the molecular packing, as in the previous study.^7^ With this planar structure, **IDIDF** crystallized in the *P*1 space group of the triclinic system, with unit cell dimensions of *a* = 5.5351(1) Å, *b* = 13.7138(2) Å, *c* = 17.7912(3) Å, *α* = 68.191(1)°, *β* = 82.836(1)°, and *γ* = 80.889(1)°. As previously reported, *p*,*p*-spiro-OMeTAD exhibited a non-coplanar molecular structure and no direct short contact within the unit cell, thereby preventing π–π overlap.^3*j*^ In contrast, as shown in Fig. 3c, **IDIDF** molecules stacked toward the *a*-axis with a slipped π-stacking motif, and the estimated π–π distance was 3.480 Å. Interestingly, one **IDIDF** has multiple short contacts with the neighbouring 8 molecules, and beside π–π contacts (3.362 Å for C–C), all of the four aliphatic side chains of **IDIDF** participate in intermolecular interactions with short contacts (2.388 Å and 2.312 Å for H–H and 2.985 Å for S–H, see Fig. 3d), giving rise to rigid and stable crystalline networks. Considering this tight π–π stacking and multiple contacts of **IDIDF**, we could expect efficient formation of hole-carrier transport channels in a pinhole-free crystalline film state (see Fig. S3†).^9^

**Fig. 4:**
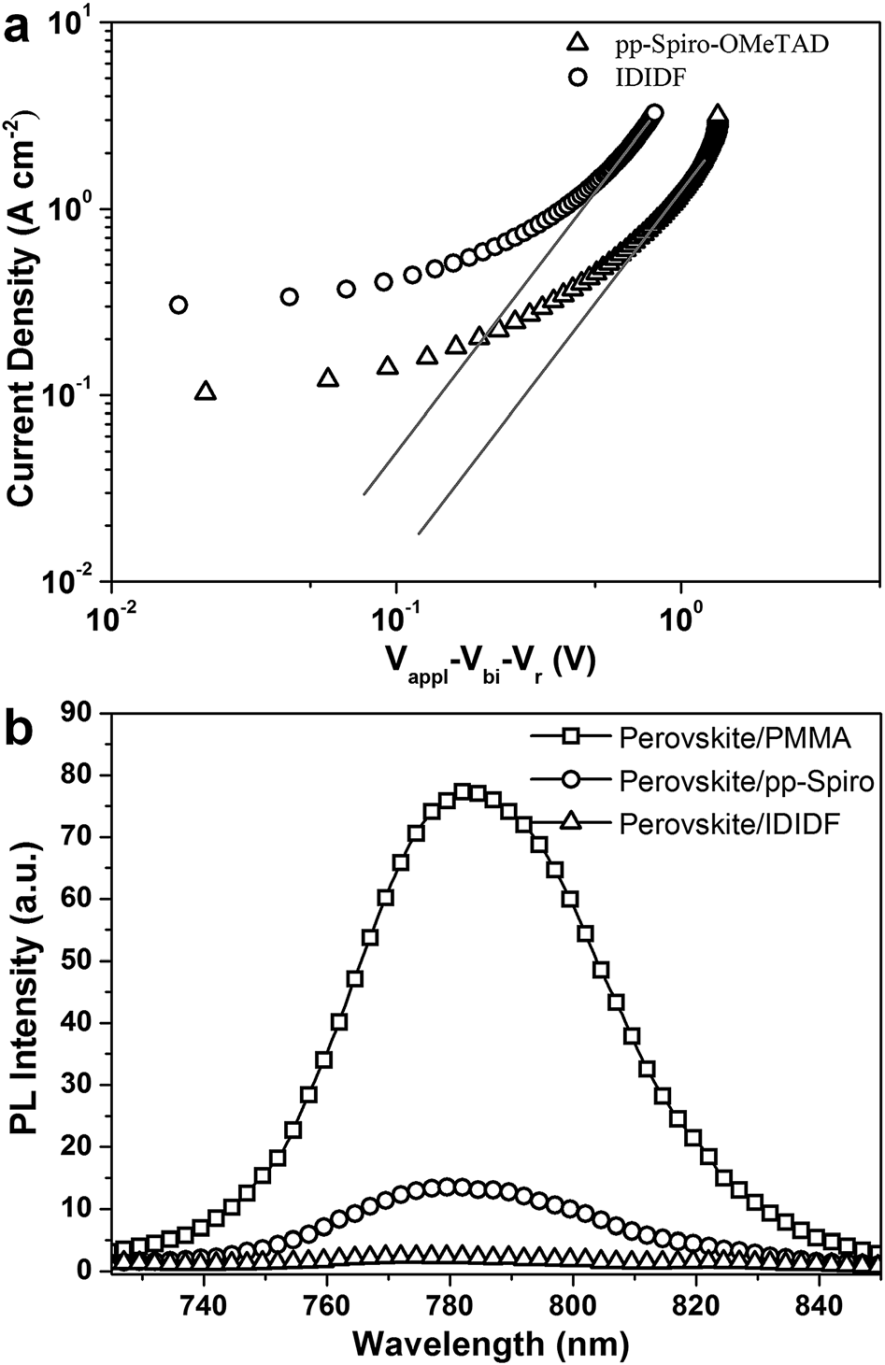
(a) The space-charge-limited-current (SCLC) of hole-only devices with the configuration of indium tin oxide (ITO)/poly(3,4-ethylenedioxythiophene)–polystyrene sulfonate (PEDOT:PSS)/*p*,*p*-spiro-OMeTAD or **IDIDF** (with Li-bis(trifluoromethanesulfonyl)imide (Li-TFSI) and 4-*tert*-butylpyridine (*t*BP) as additives)/Au. (b) PL emission spectra of perovskite/PMMA, perovskite/*p*,*p*-spiro-OMeTAD, and perovskite/**IDIDF** film under excitation at 670 nm.

In order to obtain more insight into the charge transport properties of **IDIDF**, we carried out space-charge-limited currents (SCLCs) measurement according to a literature method.^10^ As shown in Fig. 4a, the hole mobility of each material was determined by fitting the *J*–*V* curves to the reported equation (see ESI†); the evaluated hole mobilities of *p*,*p*-spiro-OMeTAD and **IDIDF** are 2.17 × 10^–4^ cm^2^ V^–1^ s^–1^ and 1.69 × 10^–3^ cm^2^ V^–1^ s^–1^, respectively. The value obtained in this work for *p*,*p*-spiro-OMeTAD is similar to the data previously reported in the literature.^11^ Obviously, **IDIDF** shows a higher mobility than that of *p*,*p*-spiro-OMeTAD, which is attributed to a strong π–π interaction between the planar structures of the extended core, as revealed in Fig. 3.

Photoluminescence (PL) quenching of the perovskite emission was examined to investigate the hole-accepting capability of the HTMs in bilayered film. A perovskite film employing a mixed perovskite of FAPbI_3_ and MAPbBr_3_ was prepared according to our previous publication.^1c,e^ Upon excitation at 670 nm, a broad PL band of perovskite/PMMA film is observed to be centered at 780 nm. Perovskite/**IDIDF** film shows a much larger decrease in PL intensity, as compared with perovskite/*p*,*p*-spiro-OMeTAD film. This means that the charge transfer is more effective at the interface with **IDIDF**, exhibiting a better hole collection capability at the perovskite/**IDIDF** interface.^3g,3f,12^

With respect to the electrochemical properties, we recorded a cyclic voltammogram using the solid film, as shown in Fig. S4.† From this measurement, the HOMO energy levels were estimated to be –5.23 eV and –4.93 eV for **IDIDF** and *p*,*p*-spiro-OMeTAD, respectively. Their optical band gaps were found to be 2.30 eV and 2.94 eV from the edge of the absorption spectra, and thus the LUMO energy levels were evaluated to be –2.93 eV and –1.99 eV, respectively. In comparison to *p*,*p*-spiro-OMeTAD, the HOMO energy level of **IDIDF** was lower, but was still higher than the valence band (5.3 eV) of the perovskite, thereby providing sufficient driving force for hole transfer at the interface of the perovskite and **IDIDF** (see Fig. S5†). Importantly, however, this lower HOMO level is expected to lead to a higher open-circuit voltage (*V*_oc_) for **IDIDF**.

**Fig. 5:**
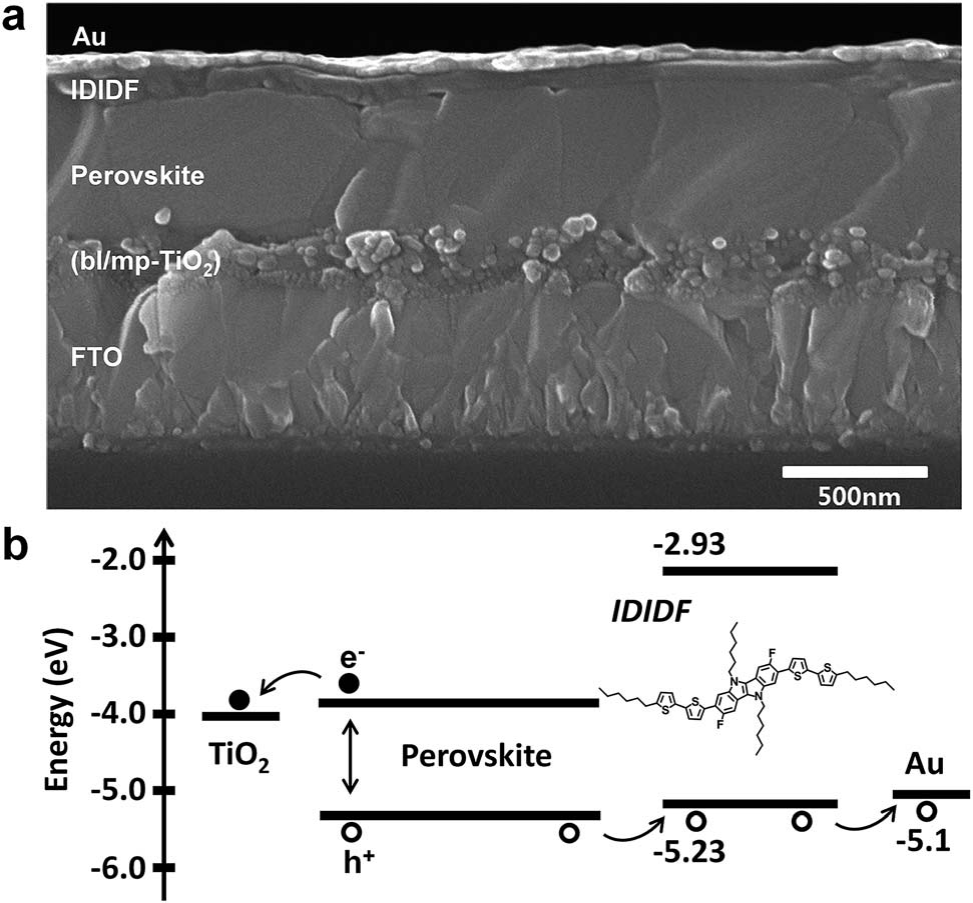
(a) Cross-sectional scanning electron microscopy (SEM) image of the device including FTO/bl-TiO_2_/mp-TiO_2_/perovskite/**IDIDF**/Au. (b) The schematic energy diagram of the corresponding device.

We fabricated FAPbI_3_-based PSC, which is based on a typical bilayered device configuration consisting of a fluorine-doped tin oxide (FTO) substrate/blocking layer, (bl)-TiO_2_/mp-TiO_2_/FAPbI_3_-based perovskite/HTM/Au. Through our solvent engineering technique, we prepared a dense and flat perovskite-absorbing layer on the mp-TiO_2_ scaffold, as shown in a cross-sectional SEM image of the whole device (see Fig. 5a). The subsequent deposition of **IDIDF** forms a ∼100 nm thick HTM layer, which is clearly distinguished from the perovskite layer and the Au electrode. Details are added in the ESI.† Fig. 5b shows a schematic energy-level diagram of the whole device. As explained above, **IDIDF** has appropriate HOMO and LUMO levels for facilitating hole extraction and blocking electron leakage, considering the valence and conduction bands of the perovskite in the device.

**Fig. 6:**
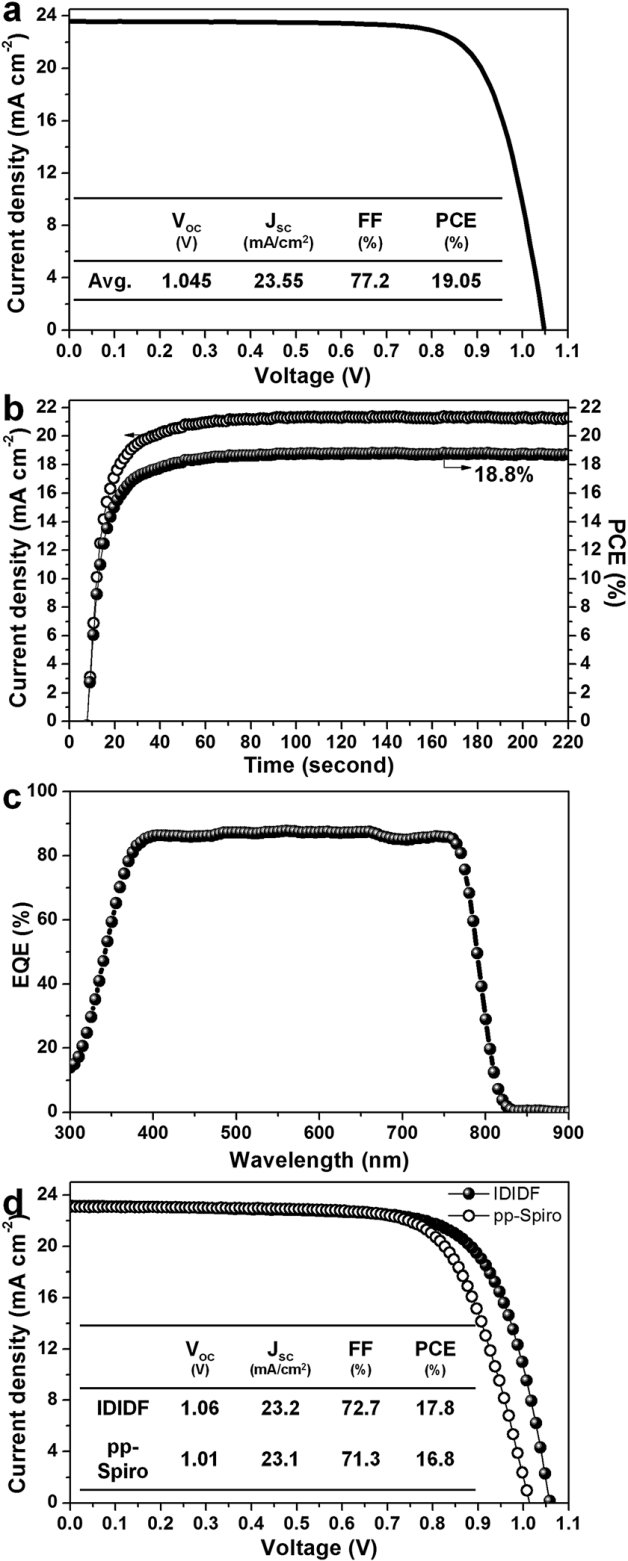
(a) Current density–voltage (*J*–*V*) curves of the best device (FTO/bl-TiO_2_/mp-TiO_2_/perovskite/**IDIDF**/Au). (b) The steady-state photocurrent and efficiency of the corresponding device at the maximum power point (0.88 V). (c) The corresponding external quantum efficiency (EQE) spectra. (d) A comparison of *J*–*V* curves of the devices fabricated using **IDIDF** and commercial *p*,*p*-spiro. Li-bis(trifluoromethanesulfonyl)imide (Li-TFSI) and 4-*tert*-butylpyridine (*t*BP) are used as additives in this work. Statistical power conversion efficiency distributions of 32 PSC devices for **IDIDF** and *p*,*p*-spiro-OMeTAD are depicted in Fig. S6.†

Fig. 6a presents the average current density–voltage (*J*–*V*) curves for the best photovoltaic device fabricated using **IDIDF** under reverse and forward scans, resulting in an average PCE of 19.05%, together with *J*_sc_ = 23.55 mA cm^–2^, *V*_oc_ = 1.045 V, and FF = 77.2%. The curve values obtained in both scan directions with 10 mV voltage steps and a delay time of 50 ms are averaged; the device shows a PCE of 19.8% for a reverse scan (from *V*_oc_ to *J*_sc_), and a PCE of 18.3% for a forward scan (from *J*_sc_ to *V*_oc_) (see Fig. S7 in ESI†). To obtain the exact efficiency of the resultant device, the steady-state photocurrent was monitored at a maximum power point of 0.88 V and the corresponding steady-state efficiency was estimated to be 18.8% (see Fig. 6b). This is close to the average PCE in the device. An external quantum efficiency (EQE) spectrum for the resultant device is shown in Fig. 6c. The integrated *J*_sc_ calculated from the EQE spectrum (∼23 mA cm^–2^) is similar to the measured *J*_sc_. A high EQE of 80–88% in a broad range from 370 to 770 nm was found, which indicates efficient light harvesting originating from a light absorber of the FAPbI_3_-based perovskite, thereby contributing to a high *J*_sc_. To unambiguously compare the performance with that of *p*,*p*-spiro-OMeTAD, we prepared devices using **IDIDF** and *p*,*p*-spiro-OMeTAD under the same conditions. As shown in Fig. 6d, the device fabricated using **IDIDF** exhibits a higher PCE than that using *p*,*p*-spiro-OMeTAD. Especially, as compared to other photovoltaic parameters, a relatively meaningful difference in *V*_oc_ is found, which is supported by the fact that the HOMO energy level of **IDIDF** was lower than that of *p*,*p*-spiro-OMeTAD. Furthermore, as compared to *p*,*p*-spiro-OMeTAD, the higher hole mobility and higher hole collection capability of **IDIDF** must have facilitated hole extraction toward the Au, while minimizing carrier recombination, which is one of the factors leading to a higher FF and PCE. Finally, we tested the long-term stability of the device without encapsulation under a high humidity of 85%. For the *p*,*p*-spiro-OMeTAD-containing device, the performance decreased rapidly after exposure to moisture. When the device was exposed for 50 h, it became severely damaged. In contrast, the **IDIDF**-containing device showed a much better stability. The efficiency was maintained at almost 90% of its initial value after 50 h, although the HTM contained hygroscopic additives (Li-TFSI and tBP). It is noted that **IDIDF** molecules with highly crystalline packing act as a more effective moisture barrier than *p*,*p*-spiro-OMeTAD, with an amorphous glass nature (see Fig. S8†).

## Conclusion

We synthesized a fluorinated indoloindole derivative as a high-performance crystalline HTM for perovskite solar cells. A planar π-conjugated backbone linked with a flexible alkyl chain enabled the formation of a molecular arrangement stacked by strong π–π interactions, which was revealed in single-crystal analysis. In this regard, **IDIDF** in the film state showed a higher mobility than that of *p*,*p*-spiro-OMeTAD. PL quenching occurred more effectively at the perovskite/**IDIDF** interface, compared to that at the perovskite/*p*,*p*-spiro-OMeTAD. From CV measurement, proper HOMO and LUMO energy levels for **IDIDF** were found to be suitable for a HTM. As a result, the device fabricated using **IDIDF** showed a better performance as compared to *p*,*p*-spiro-OMeTAD, exhibiting a best PCE of 19%. It was thus shown that a planar IDID core-based crystalline HTM is a promising candidate for highly efficient perovskite solar cells.

## Experimental

### Synthetic procedures

**IDIDF** was synthesized according to the procedure shown in Scheme 1. Unless stated otherwise, all reagents were purchased at Sigma Aldrich, TCI, and Alfa Aesar.

#### Synthesis of 5-fluoro-2-nitroaniline (**2**)

5-Fluoro-2-nitrobenzene (30.00 g, 192.16 mmol), *N*-bromosuccinimide (35.91 g, 201.77 mmol), and 500 mL of acetic acid (AcOH) were added to a 1000 mL two-necked round-bottom flask (RBF), equipped with a magnetic stirrer bar and a reflux condenser. The reaction mixture was gently refluxed for 1.5 hours. After the reaction finished, the reaction mixture was cooled down to room temperature, then poured into 1500 mL of H_2_O. The yellow precipitates were collected by filtration and washed with H_2_O (500 mL × 2) to afford **2** (40.35 g, yield 89.35%). ^1^H-NMR (300 MHz, CDCl_3_, *δ*): 8.39 (d, *J* = 7.08 Hz, 1H), 6.60 (d, *J* = 9.60 Hz, 1H), 6.1963 (s, 2H).

#### Synthesis of 1-bromo-2-fluoro-4-iodo-5-nitrobenzene (**3**)

An H_2_O (40 mL) and H_2_SO_4_ (75 mL) mixed solution was slowly added to a mixed solution of AcOH (70 mL), and **2** (20.00 g, 85.10 mmol), using a dropping funnel at 0 °C. Afterwards, NaNO_2_ solution (6.46 g, 93 mmol, in 20 mL of H_2_O) was added dropwise to the reaction mixture and stirred for an hour. KI solution (16.95 g, 102 mmol, in 20 mL of H_2_O) was then added dropwise to the reaction mixture. After completion of the addition, the reaction mixture was heated to 60 °C for 3 hours, cooled down to 0 °C, and methylene chloride (DCM) was added until all the precipitates were completely dissolved. The reaction mixture was poured into 400 mL of saturated NaHCO_3_ aqueous solution in an ice bath and extracted with DCM. The organic phase was washed with brine and saturated Na_2_S_2_O_3_ aqueous solution (250 mL × 2, each), dried over MgSO_4_, filtered, and concentrated, sequentially. The concentrated crude product was recrystallized from hexane to afford **3** as an orange crystal (15.70 g, yield 53.35%). ^1^H-NMR (300 MHz, CDCl_3_, *δ*): 8.18 (d, *J* = 6.21 Hz 1H), 7.81 (d, *J* = 7.35 Hz, 1H).

#### Synthesis of ((4-bromo-5-fluoro-2-nitrophenyl)ethynyl)trimethylsilane (**4**)

**3** (7 g, 20.24 mmol), bis(triphenylphosphine)palladium(ii) dichloride (710 mg, 1.01 mmol), and copper(i) iodide (385 mg, 2.02 mmol) were added to a 100 mL two-necked RBF, equipped with a magnetic stirrer bar. The reaction vessel was evacuated and backfilled with argon (Ar) gas. Then, THF (40 mL), trimethylsilylacetylene (1.99 g, 20.24 mmol), and triethylamine (12 mL) were added. After 3 hours stirring at room temperature, the reaction mixture was filtered through a silica plug. The concentrated filtrate was purified by column chromatography (ethyl acetate (EA)/*n*-hexane 1 : 9, v/v) to afford **4** as a dark brown oil (4.85 g, yield 75.79%). ^1^H-NMR (300 MHz, CDCl_3_, *δ*): 8.31 (d, *J* = 6.24 Hz, 1H), 7.38 (d, *J* = 8.19 Hz, 1H), 0.28 (s, 9H).

#### Synthesis of 1-bromo-4-ethynyl-2-fluoro-5-nitrobenzene (**5**)

**4** (4.8 g, 15.33 mmol), K_2_CO_3_ (2.33 g, 16.86 mmol), 18-crown-6 (0.41 g 1.53 mmol), and a mixed-solvent of methylene chloride (30 mL), H_2_O (15 mL), and acetone (7 mL) were added to a 100 mL one-necked RBF, equipped with a magnetic stirrer bar. After 2 hours stirring at room temperature, the reaction mixture was poured into H_2_O (300 mL), and extracted with DCM. The organic phase was separated, washed with brine (300 mL × 2), dried over MgSO_4_, and concentrated, sequentially. The concentrated crude product was purified by column chromatography (EA/*n*-hexane 1 : 3, v/v) to afford **5** as a brown powder (2.79 g, yield 75.57%). ^1^H-NMR (300 MHz, CDCl_3_, *δ*): 8.36 (d, *J* = 6.24 Hz, 1H), 7.43 (d, *J* = 8.04 Hz, 1H), 3.64 (s, 1H).

#### Synthesis of 1,2-bis(4-bromo-5-fluoro-2-nitrophenyl)ethyne (**6**)

Compound **6** was synthesized by the same synthetic procedure as compound **4**, using compound **3** (4.80 g, 13.89 mmol), compound **5** (3.39 g, 13.89 mmol), bis(triphenylphosphine)palladium(ii) dichloride (487 mg, 0.69 mmol), copper(i) iodide (264 mg, 1.38 mmol), THF (40 mL), and triethylamine (10 mL). Flash column chromatography (CHCl_3_) and methanol washing (100 mL × 1) afforded compound **6** as a brown solid (2.90 g, yield 45.18%). ^1^H-NMR (300 MHz, CDCl_3_, *δ*): 8.47 (d, *J* = 6.18 Hz, 2H), 7.56 (d, *J* = 7.86 Hz, 2H).

#### Synthesis of 1,2-bis(4-bromo-5-fluoro-2-nitrophenyl)ethane-1,2-dione (**7**)

Potassium permanganate (2.19 g, 13.84 mmol), Adogen 464 (catalytic amount), H_2_O (30 mL), DCM (40 mL), and AcOH (1.5 mL) were added to a 250 mL two-necked RBF, equipped with a magnetic stirrer bar. The reaction mixture was stirred, and evacuated and backfilled with Ar. Then, **6** (2.13 g, 4.64 mmol) was added to the reaction mixture. The reaction mixture was gently refluxed for 5 hours, cooled, and decolorized using NaHSO_3_, sequentially. The resulting two clear phases were separated, and the yellow organic phase was dried over MgSO_4_ and filtered through a silica plug. The yellow filtrate was concentrated, and the resulting solid was washed with methanol to afford **7** as a yellow crystalline solid (1.85 g, yield 81.23%). ^1^H-NMR (300 MHz, CDCl_3_, *δ*): 8.55 (d, *J* = 5.61 Hz, 2H), 7.4 (d, *J* = 6.93 Hz, 2H).

#### Synthesis of 2,7-dibromo-3,8-difluoro-5,10-dihydroindolo[3,2-*b*]indole (**8**)

**7** (1.85 g, 3.74 mmol), and AcOH (20 mL) were added to a 100 mL two-necked RBF, equipped with a magnetic stirrer bar. With vigorous stirring, the filtrate of stannous chloride (14.87 g, 74.89 mmol), acetic acid (15 mL), and 1 N HCl (15 mL) mixed solution was added to the reaction mixture. The reaction mixture was gently refluxed for 5 hours at 80 °C, and cooled down to room temperature. After that, the reaction mixture was poured into H_2_O (300 mL), and extracted with EA. The concentrated crude product was purified by flash column chromatography (EA/*n*-hexane 1 : 2 v/v) and the resulting solid was washed with chloroform (100 mL × 1) to afford **8** as a light brown solid (1.23 g, yield 82.10%). ^1^H-NMR (300 MHz, acetone-d_6_, *δ*): 10.47 (s, 2H), 7.84 (d, *J* = 5.88 Hz, 2H), 7.67 (d, *J* = 9.36 Hz, 2H).

#### Synthesis of 2,7-dibromo-3,8-difluoro-5,10-dihexyl-5,10-dihydroinolo[3,2-*b*]indole (**9**)

A 100 mL two-necked RBF, equipped with a magnetic stirrer bar and reflux condenser was baked under reduced pressure and backfilled with Ar (×3). Compound **8** (380 mg, 0.95 mmol), anhydrous THF (20 mL), and NaH (91.20 mg, 3.79 mmol) were added to the baked reaction vessel. After 10 minutes at room temperature, 1-bromohexane (627 mg, 3.79 mmol) was added to the reaction mixture. After that, the reaction mixture was gently refluxed for 24 hours. After the reaction was completed, the reaction mixture was poured into brine (200 mL) and extracted with DCM. The organic layer was separated, washed (water, 200 mL × 3), dried with MgSO_4_, and concentrated, sequentially. The resulting crude product was purified by flash column chromatography (EA/*n*-hexane 1 : 4, v/v) and recrystallization (EA) to afford **9** as a white crystalline solid (410 mg, yield 75.94%). ^1^H-NMR (300 MHz, THF-d_8_, *δ*): 7.94 (d, *J* = 5.76 Hz, 2H), 7.84 (d, *J* = 9.45 Hz, 2H), 4.62 (t, *J* = 7.08 Hz, 4H), 1.94 (m, *J* = 7.23 Hz, 4H), 1.45–1.19 (m, 12H), 0.81 (t, *J* = 7.11 Hz, 6H).

#### Synthesis of **IDIDF**

**9** (650 mg, 1.14 mmol), 5′-hexyl-2,2′-bithiophene-5-boronic acid pinacol ester (904 mg, 2.40 mmol), tetrakis(triphenylphosphine)palladium(0) (132 mg, 0.11 mmol), THF (30 mL), and 2 N NaOH aqueous solution (15 mL) were added to a 100 mL two-necked RBF, equipped with a magnetic stirrer bar and reflux condenser. The reaction mixture was gently refluxed at 80 °C in an Ar atmosphere. After 24 hours, the reaction mixture was quenched with H_2_O (300 mL), neutralized (1 N HCl), and extracted with DCM. The combined organic phase was dried with MgSO_4_ and concentrated under reduced pressure. The crude product was purified by flash column chromatography (chloroform/*n*-hexane 1 : 4, v/v) and recrystallization (EA) to afford **IDIDF** as orange crystals (760 mg, yield 73.23%). ^1^H-NMR (500 MHz, tetrahydrofuran-d_8_, *δ*): 7.78 (d, *J* = 6 Hz, 2H), 7.69 (d, *J* = 12 Hz, 2H), 7.46 (d, *J* = 3.5 Hz, 2H), 7.16 (d, *J* = 4 Hz, 2H), 7.07 (d, *J* = 3.5 Hz, 2H), 6.74 (d, *J* = 3.5 Hz, 2H), 4.58 (t, *J* = 7 Hz, 4H), 2.82 (t, *J* = 7.5 Hz, 4H), 1.98–1.93 (m, 4H), 1.71–1.69 (m, 4H), 1.45–1.27 (m, 24H), 0.91 (t, *J* = 7 Hz, 6H), 0.85 (t, *J* = 7 Hz, 6H). ^13^C NMR (500 MHz, tetrahydrofuran-d_8_, *δ*): 155.73, 153.83, 146.13, 139.27, 138.48, 138.45, 138.25, 138.22, 135.95, 129.09, 127.24, 127.20, 126.04, 124.37, 124.14, 118.07, 117.94, 114.01, 113.92, 109.36, 109.33, 104.99, 104.78, 45.98, 32.74, 31.38, 31.01, 29.84, 27.70, 23.64, 23.60, 14.59, 14.52, HRMS (FAB, *m*/*z*): calcd for C_54_H_62_F_2_N_2_S_4_: 906.39, found: 906.392. Elem. anal. calcd for C_54_H_62_F_2_N_2_S_4_: C 71.48, H 7.11, F 4.19, N 3.09, S 14.13; found: C 71.14, H 7.22, N 3.08, S 13.94.

### Device fabrication and measurement

The F-doped SnO_2_ (FTO, Pilkington, TEC8) substrate was cleaned in an ultrasonic bath containing detergents for 30 min, and then a dense blocking layer of TiO_2_ (60 nm, bl-TiO_2_) was deposited onto the FTO by spray pyrolysis, using a 20 mM titanium diisopropoxide bis(acetylacetonate) solution (Aldrich) at 450 °C. A 100 nm thin mesoporous (mp)-TiO_2_ was spin-coated on top of the bl-TiO_2_/FTO substrate at 1000 rpm for 50 s, using home-made TiO_2_ (∼50 nm in particle size) pastes. The pristine paste had been diluted in 2-methoxyethanol (1 g/5 mL), and calcinated at 500 °C for 1 h in air, which led to a thickness of about 100 nm. The (FAPbI_3_)_0.92_(MAPbBr_3_)_0.08_ perovskite solutions with a small excess of PbI_2_ were then coated onto the mp-TiO_2_/bl-TiO_2_/FTO substrate heated to 50 °C by two consecutive spin-coating steps, at 1000 and 5000 rpm for 5 s and 10 s, respectively. During the second spin-coating step, 1 mL ethyl ether was poured onto the substrate. The 1.05 M solution for (FAPbI_3_)_0.92_(MAPbBr_3_)_0.08_ perovskite was obtained by dissolving NH_2_CH=NH_2_I (=FAI) and CH_3_NH_3_Br (=MABr) with PbI_2_ and PbBr_2_ in *N*,*N*-dimethylformamide (=DMF) and dimethylsulfoxide (=DMSO) = (6 : 1 v/v). Then, the perovskite-deposited substrate was dried on a hot plate at 150 °C for 10 min. A *p*,*p*-spiro-OMeTAD/chlorobenzene (30 mg/1 mL) solution with 21.5 μL Li-bis(trifluoromethanesulfonyl) imide (Li-TFSI)/acetonitrile (170 mg/1 mL) and 21.5 μL 4-*tert*-butylpyridine (TBP)/acetonitrile (1 mL/1 mL) as additives was spin-coated on the (FAPbI_3_)_0.92_(MAPbBr_3_)_0.08_/mp-TiO_2_/bl-TiO_2_/FTO substrate at 3000 rpm for 30 s. By following the same procedure, **IDIDF** was deposited. Finally, the Au counter electrode was deposited by thermal evaporation. The active area of this electrode was fixed at 0.16 cm^2^. The cross-sections of the perovskite films were investigated using FE-SEM (Tescan Mira 3 LMU FEG). The absorption spectra were obtained using a UV-visible spectrophotometer (Shimadzu UV 2550) in the wavelength range 300 nm to 850 nm. The photovoltaic properties of the devices were measured using a solar simulator (Newport, Oriel Class A, 91195 A) with a source meter (Keithley 2420) at AM 1.5 G 100 mA cm^–2^ of illumination, and a calibrated Si-reference cell certificated by NREL. The *J*–*V* curves of all the devices were measured by masking the active area using a metal mask with an area of 0.0955 cm^2^. The external quantum efficiency (EQE) was measured using a power source (Newport 300 W xenon lamp, 66920) with a monochromator (Newport Cornerstone 260) and a multimeter (Keithley 2001). The ionization energy for the mixed perovskite film on fused silica was measured using photoelectron spectroscopy (Riken Keiki AC-2).
