# Supplementary material for: Indolo[3,2-b]indole-based crystalline hole-transporting material for highly efficient perovskite solar cells
Source: Chem Sci. 2016 Sep 5;8(1):734–41. doi: 10.1039/c6sc02832b (PMC5299807; doi:10.1039/c6sc02832b)
Supplement: Supplementary file 1 [file SC-008-C6SC02832B-s001.pdf]

Electronic Supplementary Material (ESI) for Chemical Science.
This journal is © The Royal Society of Chemistry 2016

Electronic Supplementary Information (ESI)

Indolo[3,2-b]indole-based Crystalline Hole Transporting Material for Highly Efficient Perovskite Solar Cells

Illhun Cho,[a†] Nam Joong Jeon,[b†] Oh kyu Kwon,[a] Dong Won Kim,[a] Eui Hyuk Jung,[b] Jun Hong Noh,[b] Jangwon Seo,*[b] Sang Il Seok*[bc] and Soo Young Park*[a]

Experimental

1. Characterization

Chemical structures were fully identified by ^{1}H NMR (Bruker, Avance-300), ^{13C} NMR (Bruker. Avance-500), GC-MASS (JEOL, JMS-700), and elemental analysis (Flash 2000, Thermo Scientific). UV-vis spectra were recorded on a SMIMADZU UV-1650PC. Solution absorption spectra were obtained with the concentration of 1.0×10^{-5} M in THF, and film absorption spectra were obtained with spin-coated sample on quartz substrate (using 1 wt% in CHCl3, 1500rpm/60s). The out-of-plane XRD analysis was carried out using a Bruker D8-Advance X-ray diffractometer. Single crystal X-ray crystallographic data was collected by Bruker SMART APEX II X-ray diffractometer and was analyzed by Bruker SHELXTL software. HOMO energy level of the compound was obtained from the cyclic voltammetry measurement. Cyclic voltammetry measurement was performed using a 273A (Princeton Applied Research) with an one-compartment electrolysis cell consisting of a ITO patterned glass working-electrode, a platinum wire counter-electrode, and a quasi Ag^{+}/Ag electrode as reference. Measurement was performed in an acetonitrile solution with 0.1 M tetrabutylammonium tetrafluoroborate as the supporting electrolyte, at a scan rate of 50 mV/s. Oxidation potential was calibrated using ferrocene as a reference. HOMO energy level of p,p-Spiro-OMeTAD and IDIDF were evaluated from the equation of HOMO = $-(4.8+(V_{ox}-V_{ferro})$, in which V_{ox} is onset position of sample oxidation scan, V_{ferro} is onset position of ferrocene oxidation scan, and the LUMO energy level was evaluated from the HOMO energy level and the solid-state optical energy bandgap, which was obtained from the edge of absorption spectra. Hole mobilities were measured using the space-charge-limited-current (SCLC) model with hole-only device utilizing the configuration of indium tin oxide (ITO)/ Poly(3,4-ethylenedioxythiophene)-polystyrene sulfonate (PEDOT:PSS, ~30 nm)/p,p-Spiro-OMeTAD (~90 nm) or IDIDF (~90nm) (with Li-bis(trifluoromethanesulfonyl) imide (Li-TFSI) and 4-

tert-butylpyridine (*t*BP) as additives) /Au (~80 nm). The thicknesses of the coated films were measured using a surface profiler (Surfcorder ET 200, Kosaka Laboratory, Ltd.). The solution concentration of hole transport materials was identical to those used to fabricate the optimized perovskite solar cell devices (HTM solution (30mg/ml) with an additive of 21.5 μl Li-bis(trifluoromethanesulfonyl) imide (Li-TFSI)/acetonitrile (170 mg/1 ml) and 21.5 μl 4-*tert* -butylpyridine (*t*BP)/acetonitrile (1ml/1ml)). The current-voltage curve for the SCLC measurement was obtained using Keithley 4200.

According the Mott-Gurney law, hole mobilities are extrated by using the dark current under forward bias. In order to calculate hole mobility, we have utilized data from current-voltage curve region which accurately corresponds to the J-V^2 condition.

$$J = \frac{9}{8}\varepsilon_0\varepsilon_r\mu\frac{V^2}{L^3} \quad (1)$$

In this equation ε_0 is the permittivity of free space, ε_r is the dielectric constant of the organic semiconductor material (herein ε_r was assumed to be 3 that typical value for organic semiconducting material), μ is the zero-field hole mobility, V is effective voltage with the equation of $V=V_{appl}-V_{bi}-V_r$ (V_{appl}: applied bias, V_{bi}: the built in potential due to the difference in electrical contact work function, V_r: the voltage drop due to contact resistance and series resistance across the electrodes) and L is the thickness of hole transport layer. The evaluated hole mobilities of p,p-Spiro-OMeTAD and IDIDF with Li-bis(trifluoromethanesulfonyl) imide (Li-TFSI) and 4-*tert*-butylpyridine (*t*BP) as additives are 2.17×10^{-4} and 1.69×10^{-3} $cm^2v^{-1}s^{-1}$ respectively.

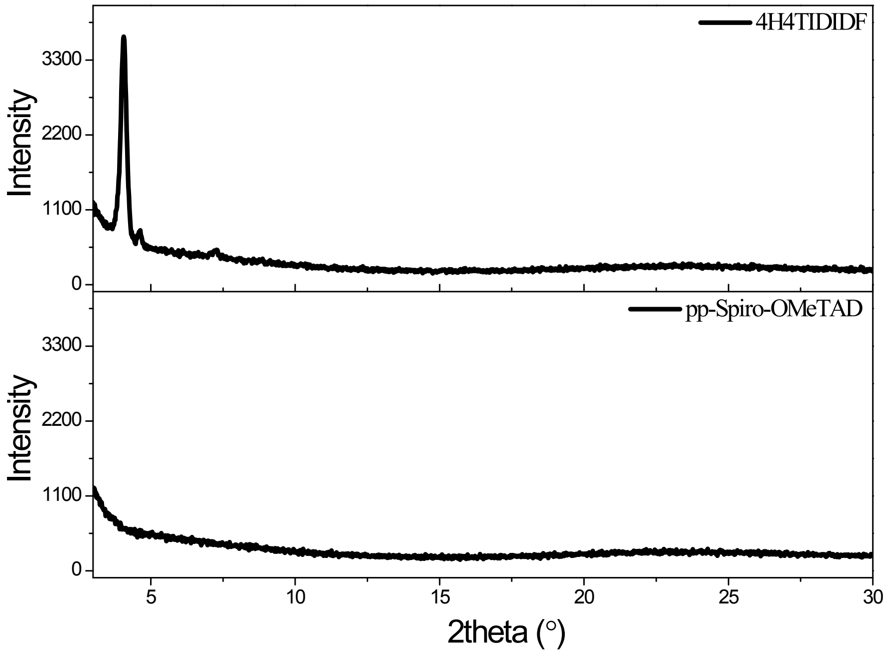

Fig. S1 Out-of-plane XRD of IDIDF and p,p-Spiro-OMeTAD.

Although, recently, crystal structure of the p,p-Spiro-OMeTAD and their melting point in powder state have been reproted,[s1-s3] the p,p-Spiro-OMeTAD film in this work, which was deposited on the perovskite layer by spin-coating process, exhibited amorphous nature.

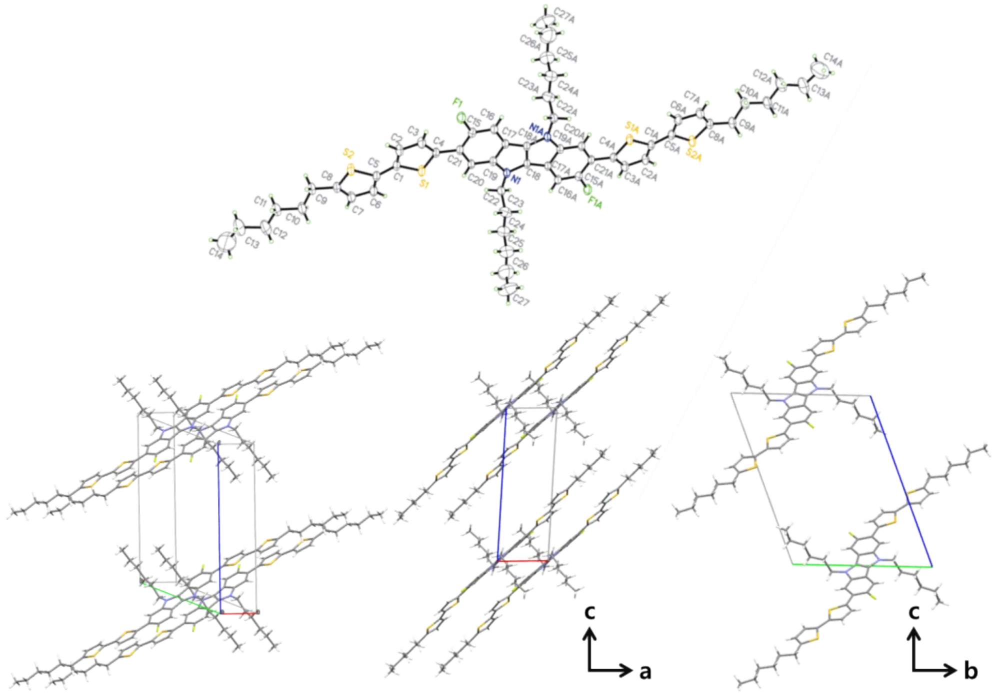

Fig. S2 Single crystal structure of IDIDF.

Identification code	20150709_0m
Empirical formula	C27 H32 F N S2
Formula weight	453.66
Temperature	296(1) K
Wavelength	0.71073 Å
Crystal system	Triclinic
Space group	P-1
Unit cell dimensions	a = 5.53520(10) Å α= 68.1910(10)°.
	b = 13.7138(2) Å β= 82.8360(10)°.
	c = 17.7912(3) Å γ = 80.8890(10)°.
Volume	1234.83(4) Å^3
Z	2
Density (calculated)	1.220 Mg/m^3
Absorption coefficient	0.237 mm^{-1}
F(000)	484
Crystal size	0.38 x 0.12 x 0.05 mm^3
Theta range for data collection	1.24 to 28.35°
Index ranges	-7<=h<=7, -16<=k<=18, 0<=l<=23
Reflections collected	6148
Independent reflections	6148 [R(int) = 0.0000]
Completeness to theta = 28.35°	99.4 %
Absorption correction	Multi-scan
Max. and min. transmission	0.9882 and 0.9152
Refinement method	Full-matrix least-squares on F^2
Data / restraints / parameters	6148 / 6 / 280
Goodness-of-fit on F^2	1.084
Final R indices [I>2sigma(I)]	R1 = 0.0756, wR2 = 0.2440
R indices (all data)	R1 = 0.1261, wR2 = 0.2884
Largest diff. peak and hole	0.619 and -0.462 e.Å^{-3}

Table S1. Crystal data and structure refinement for IDIDF.

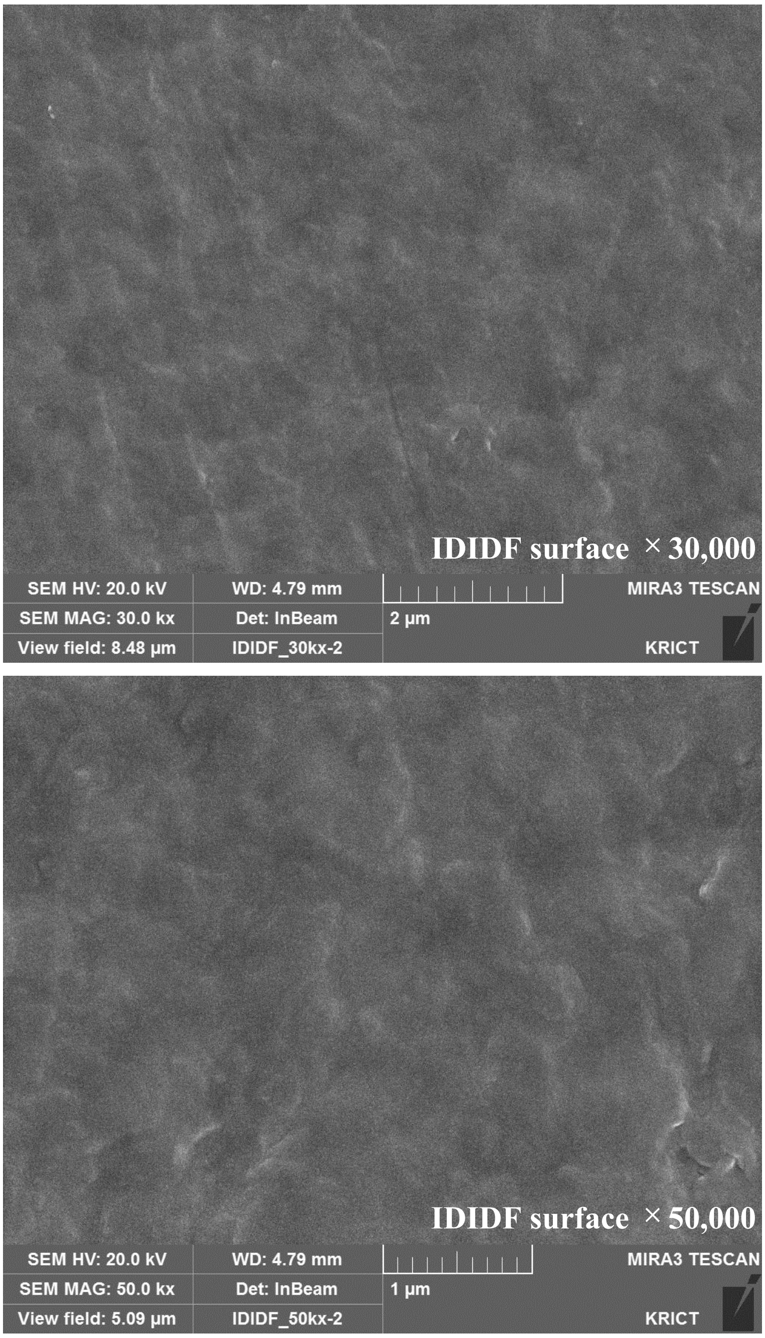

Fig. S3 **.** Higher-magnification SEM images of the top view of the IDIDF layer

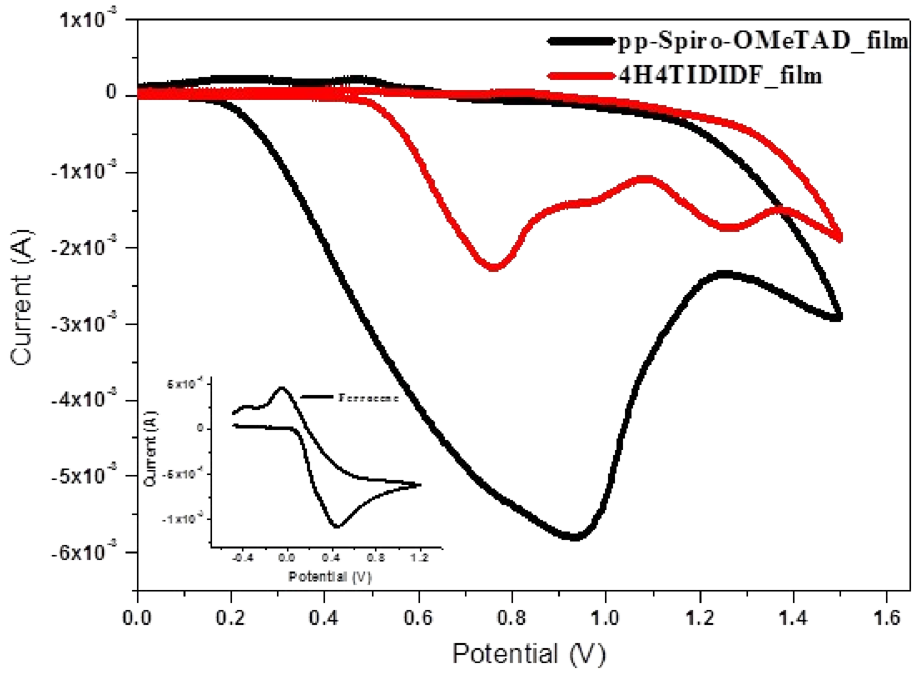

Fig. S4 Cyclic voltammograms (CVs) of IDIDF and p,p-Spiro-OMeTAD. Film samples were prepared on ITO patterned glass by drop-casting method (Inset: CV of ferrocene).

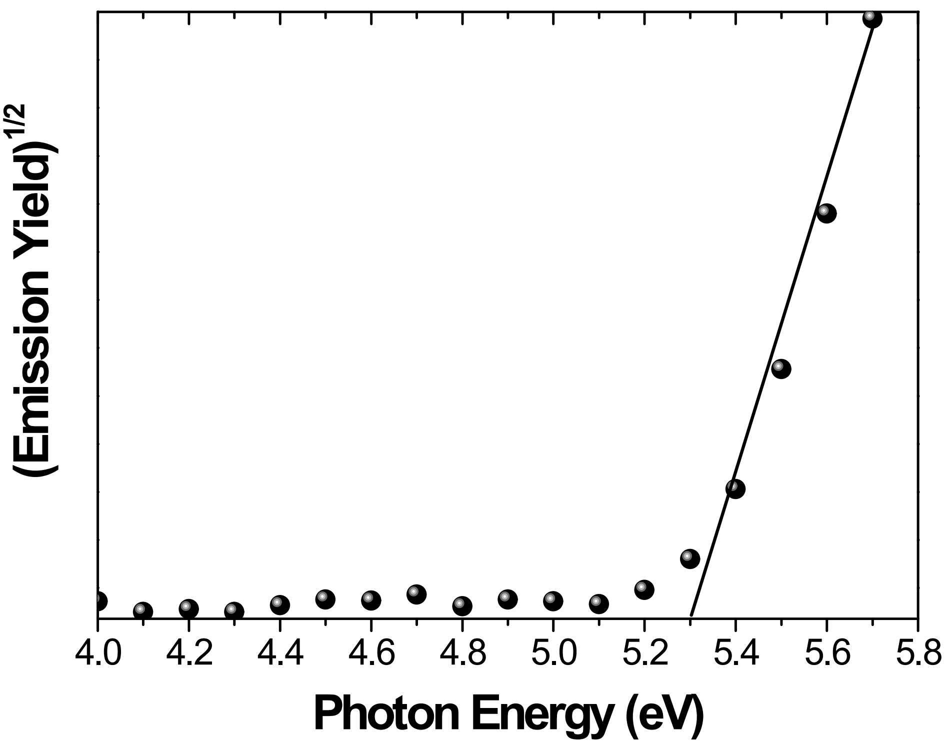

Fig. S5 Photoelectron spectrum of the mixed perovskite film on a fused silica substrate.

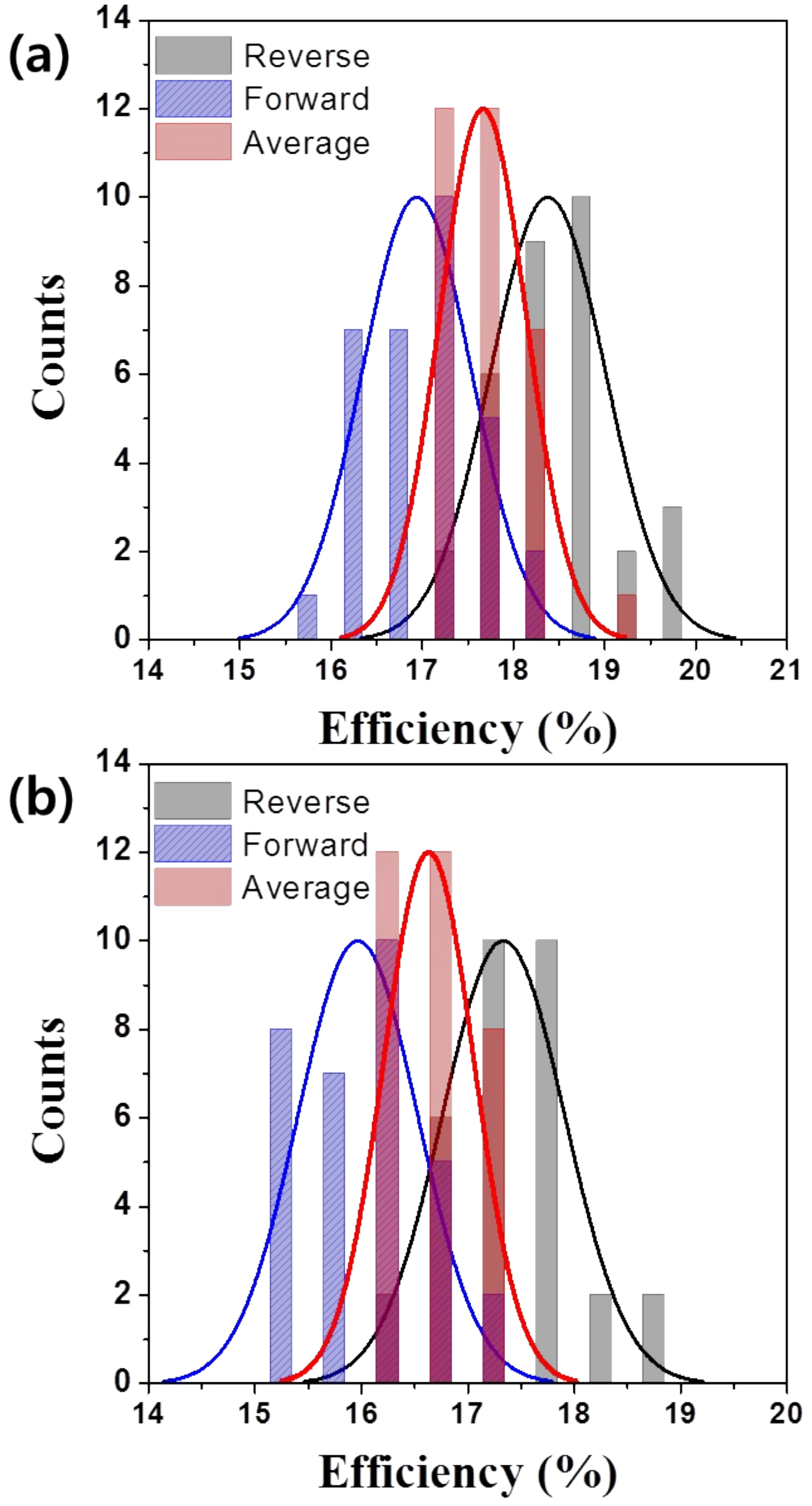

Fig. S6 Statistical power conversion efficiency distribution of 32 PSC devices for IDIDF (a) and p,p-Spiro-OMeTAD (b)

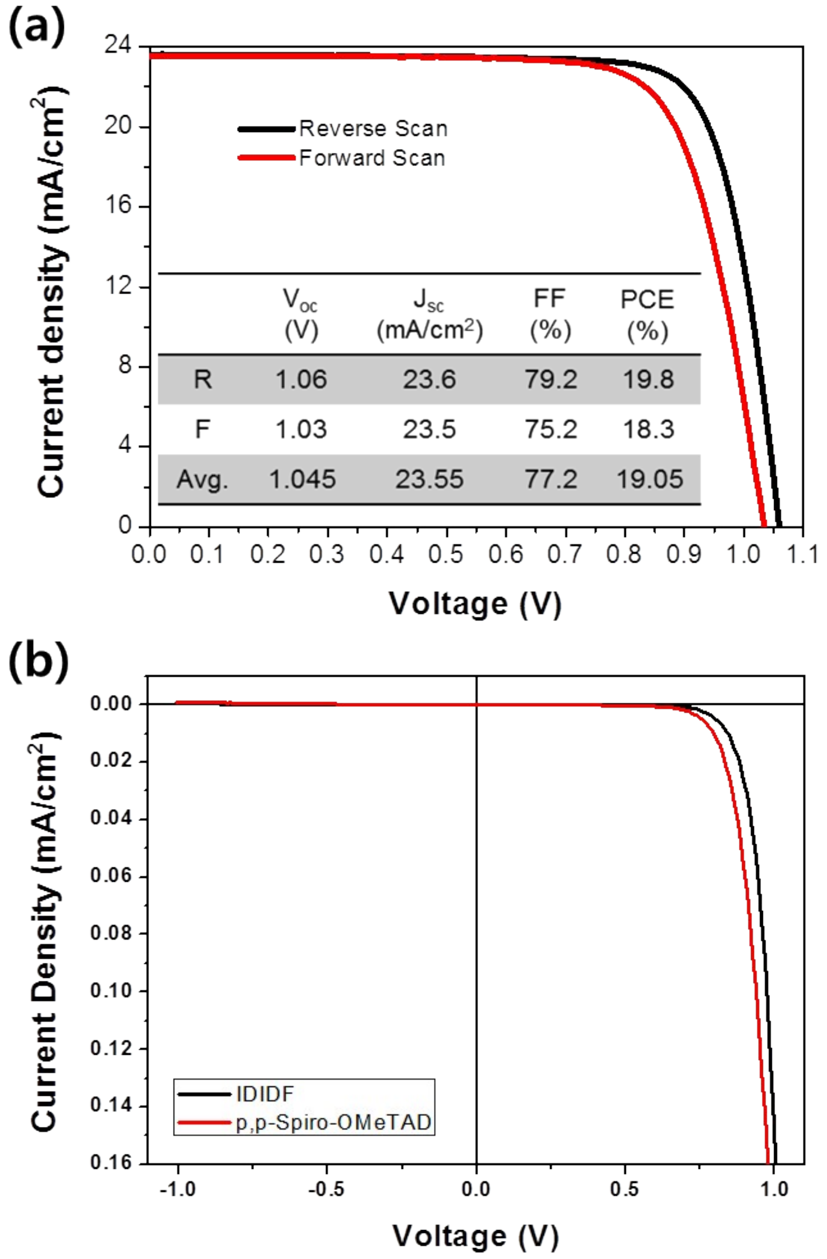

Fig. S7 (a) *J*-*V* curve for the best device with IDIDF under reverse (black) and forward (red) scans. The inset shows the average values of the photovoltaic parameters obtained from *J*-*V* curves under both scans. (b) *J*-*V* curve for dark current of the device with IDIDF (black) and p,p-Spiro-OMeTAD (red)

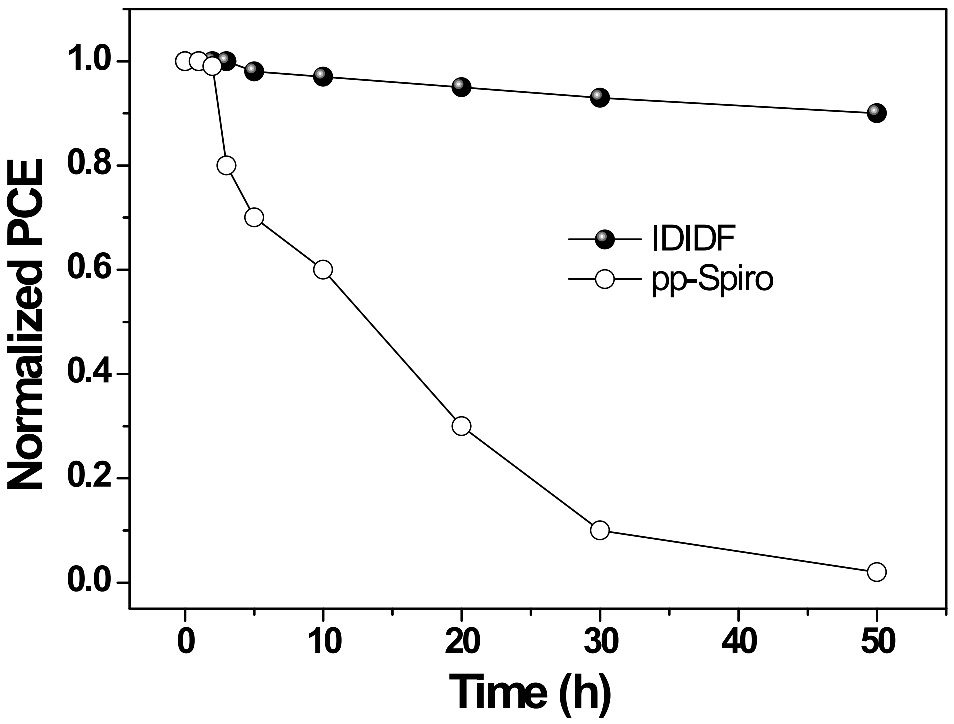

Fig. S8 Efficiency decay curves of the perovskite solar cell employing IDIDF and p,p-Spiro-OMeTAD (without encapsulation under a high humidity of 85%)

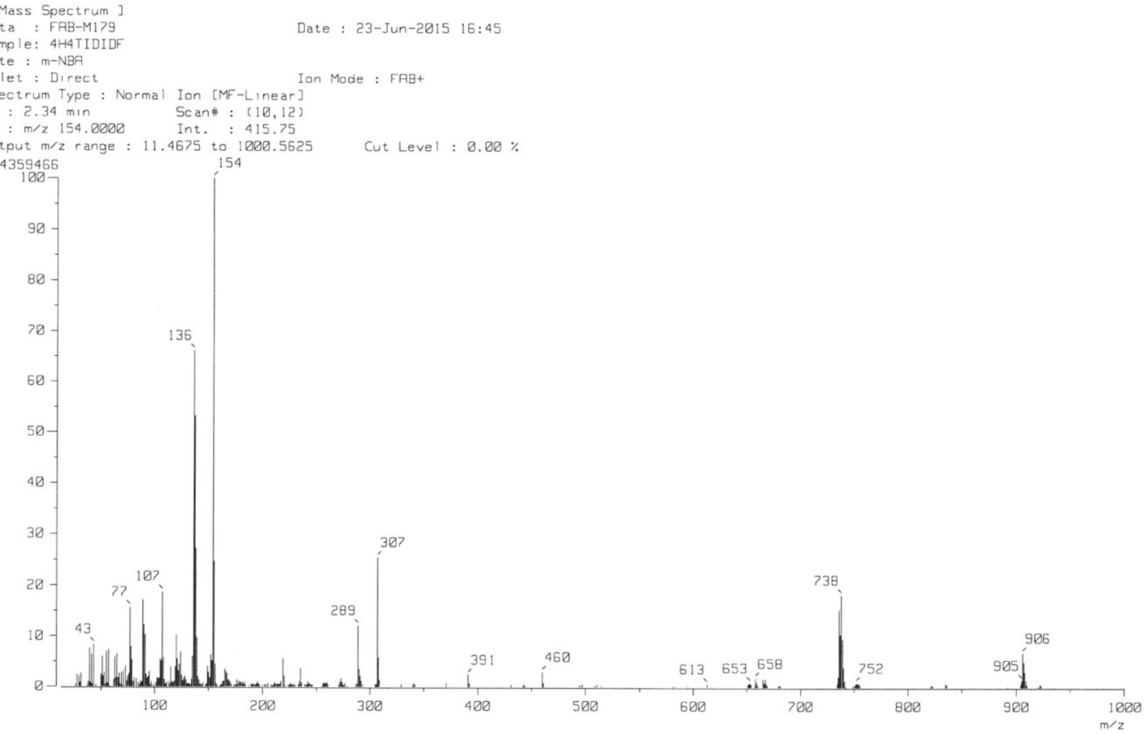

Fig. S9 high-resolution mass data of 4H4TIDIDF

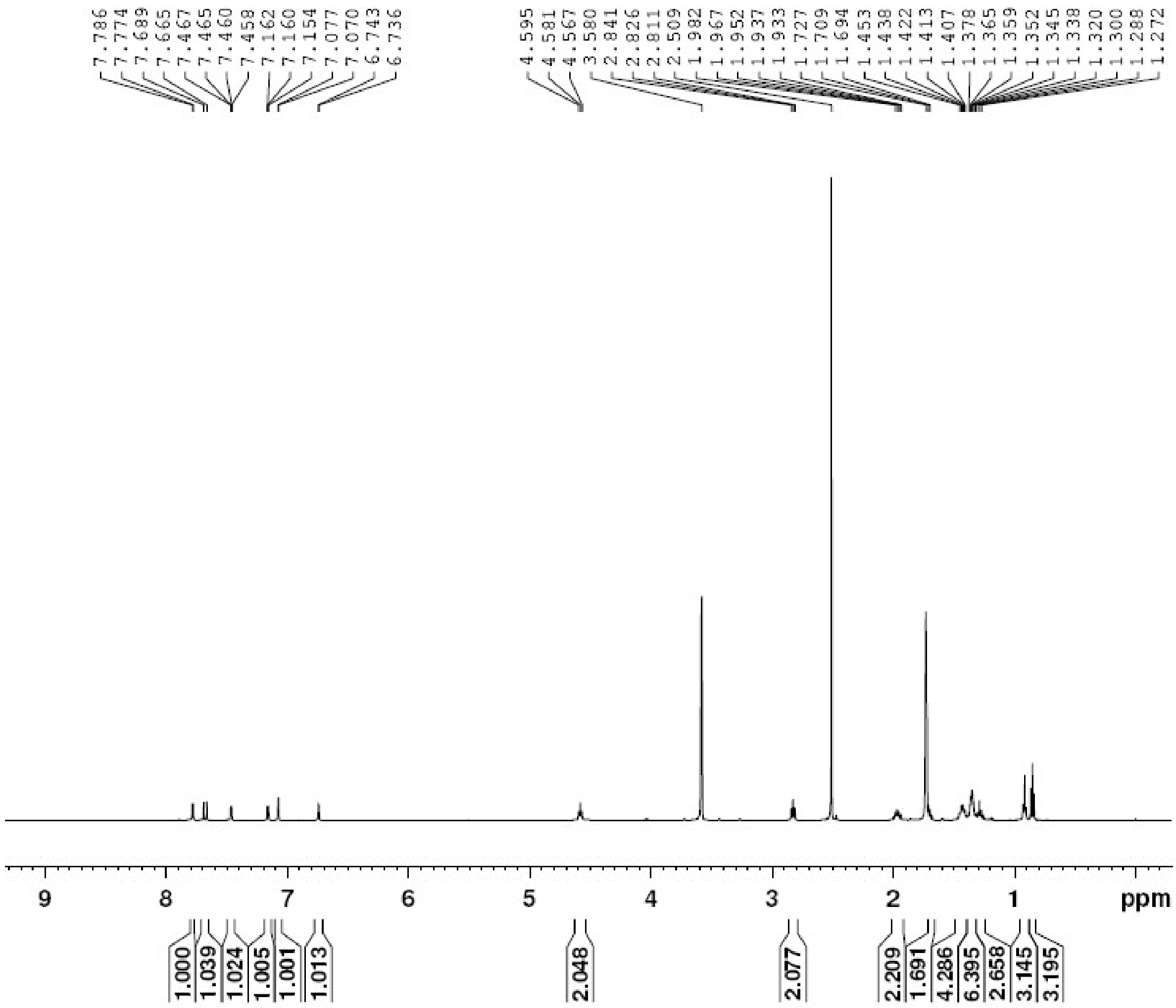

Fig. S10 ^{1}H NMR data of 4H4TIDIDF

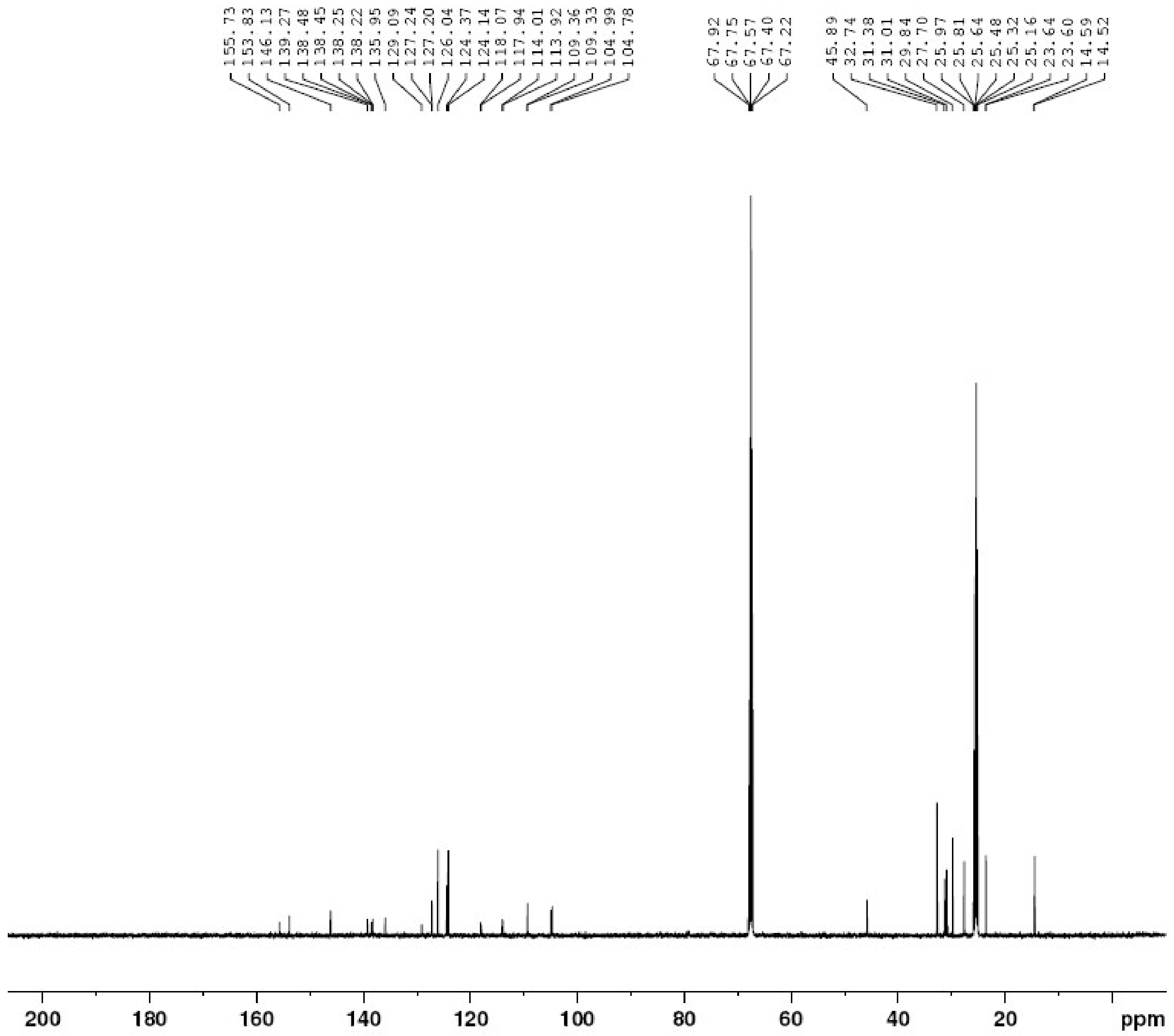

Fig. S11 ^{13}C NMR data of 4H4TIDIDF

References

s1. T. Leijten, I-K. Ding, T. Giovenzana, J. T. Bloking, M. D. MeGehee, A. Sellinger, ACS Nano, 2012, 6, 1455.

s2. K. Rakstys, M. Saliba, P. Gao, P. Gratia, E. Kamarauskas, S. Paek, V. Jankauskas, M. K. Nazeeruddin, Angew. Chem. Int. Ed. 2016, 55, 7464.

s3. P. Ganesan, K. Fu, P. Gao, I. Raabe, K. Schenk, R. Scopelliti, J. Luo, L. H. Wong, M. Grätzel, M. K. Nazeeruddin, Energy Environ. Sci. 2015, 8, 1986.
